# Simple synthesis of nanosheets of rGO and nitrogenated rGO

**DOI:** 10.3762/bjnano.11.7

**Published:** 2020-01-07

**Authors:** Pallellappa Chithaiah, Madhan Mohan Raju, Giridhar U Kulkarni, C N R Rao

**Affiliations:** 1 Centre for Nano and Soft Matter Sciences, Jalahalli, Bangalore, 560013, India; 2 New Chemistry Unit, International Centre for Materials Science, Jawaharlal Nehru Centre for Advanced Scientific Research, Jakkur P.O., Bangalore, 560064, India

**Keywords:** nanosheets, nitrogenated reduced graphene oxide (N-rGO), reduced graphene oxide (rGO), supercapacitors, thermal decomposition

## Abstract

A green and facile approach has been developed for the large-scale synthesis of nanosheets of reduced graphene oxide (rGO) and nitrogenated reduced graphene oxide (N-rGO). This has been achieved by direct thermal decomposition of sucrose and glycine at 475 °C in ca. 7 minutes, respectively. The present protocols for synthesizing rGO and N-rGO are simple and environmentally friendly as we do not use any harmful reagents, metal catalysts and solvents. Along with that, this method offers an inexpensive route with high yields to prepare rGO with a high nitrogen content (20–25 atom %). To further improve the properties of the synthesized rGO sheets, hydrogen treatment has been carried out to reduce the oxygen functional groups. Cyclic voltammograms and charge–discharge experiments have been carried out to understand the supercapacitor behavior of rGO and hydrogen treated (H-rGO) samples.

## Introduction

Graphene, the one atom thick two-dimensional material of sp^2^-hybridized carbon atoms has attracted much attention after its discovery [1–2]. It is a fascinating material used in various applications owing to its excellent electrical, optical, mechanical and thermal properties [3–5]. It has a unique electronic structure with a linear dispersion of Dirac electrons. Graphene oxide (GO) and reduced graphene oxide (rGO) are chemically modified forms of graphene, which are extensively studied in the field of science and engineering. Reduced graphene oxide has attracted significant interest due to its similarities to pristine graphene. It behaves like a semimetal or a semiconductor and is therefore used in a variety of hybrid systems such as batteries [6], electrodes [7] and photodetectors [8].

In 1958, Hummer and Offeman developed a chemical method to synthesize graphene oxide by acid treatment of graphite [9]. The graphene oxide thus obtained contains oxygen functional groups (–CO–, –COC–) on the surface and edges of the carbon sheet, which lead to a disruption of the conjugated network and the flow of charge carriers is reduced by several orders of magnitude [10]. Up to now, several methods including chemical vapor deposition [11–13], arc discharge [14], aerosol pyrolysis [15], mechanical exfoliation [1], solvothermal [16], hydrothermal synthesis [17], laser reduction of graphite oxide [18–19], and photo thermal deoxygenation of graphene oxide by camera flash have been developed to reduce the oxygen content of GO in order to restore the conjugated network [20]. Recently, a well-known chemical reduction method has been developed to obtain rGO through the reduction of exfoliated GO using various reducing agents such as hydrazine or dimethylhydrazine [21], NaBH_4_ [22], hydroquinone [23], or glucose [24]. However, these methods have not yet turned into a global strategy to prepare pure rGO in a scalable fashion. Therefore, searching for a new synthetic approach to obtain pure phases of rGO is a highly desirable and great challenge for materials chemists.

Herein, we report for the first time a generic and rapid method for the synthesis of rGO nanosheets by direct thermal decomposition of sugar, without the use of any solvents, metal catalysts, reagents and hazardous chemicals. Similarly, N-rGO nanosheets have also been synthesized using glycine as precursor.

## Results and Discussion

**Figure 1:**
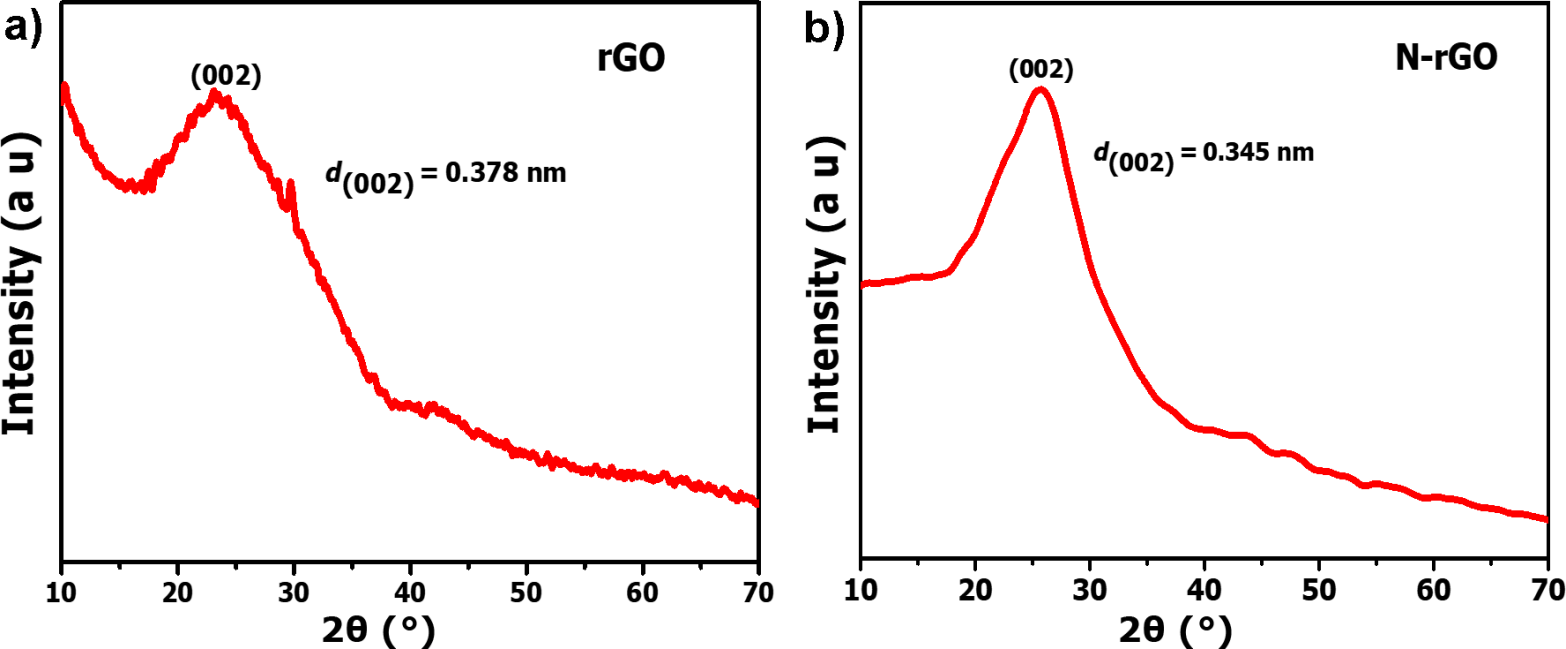
XRD patterns of (a) rGO and (b) N-rGO nanosheets.

The typical XRD patterns of rGO and N-rGO nanosheets are shown in Figure 1. The XRD pattern of the as-prepared rGO (Figure 1a) exhibits a broad peak at 23.5° corresponding to an interlayer *d*-spacing of 0.378 nm. The XRD pattern of N-rGO (Figure 1b) shows a diffraction peak at 25.8° corresponding to an interlayer d spacing of 0.345 nm. From the XRD patterns, it is observed that the peak commonly obtained for GO around 2θ of 10.3° does not appear indicating that the precursors were directly converted into rGO and N-rGO nanosheets.

**Figure 2:**
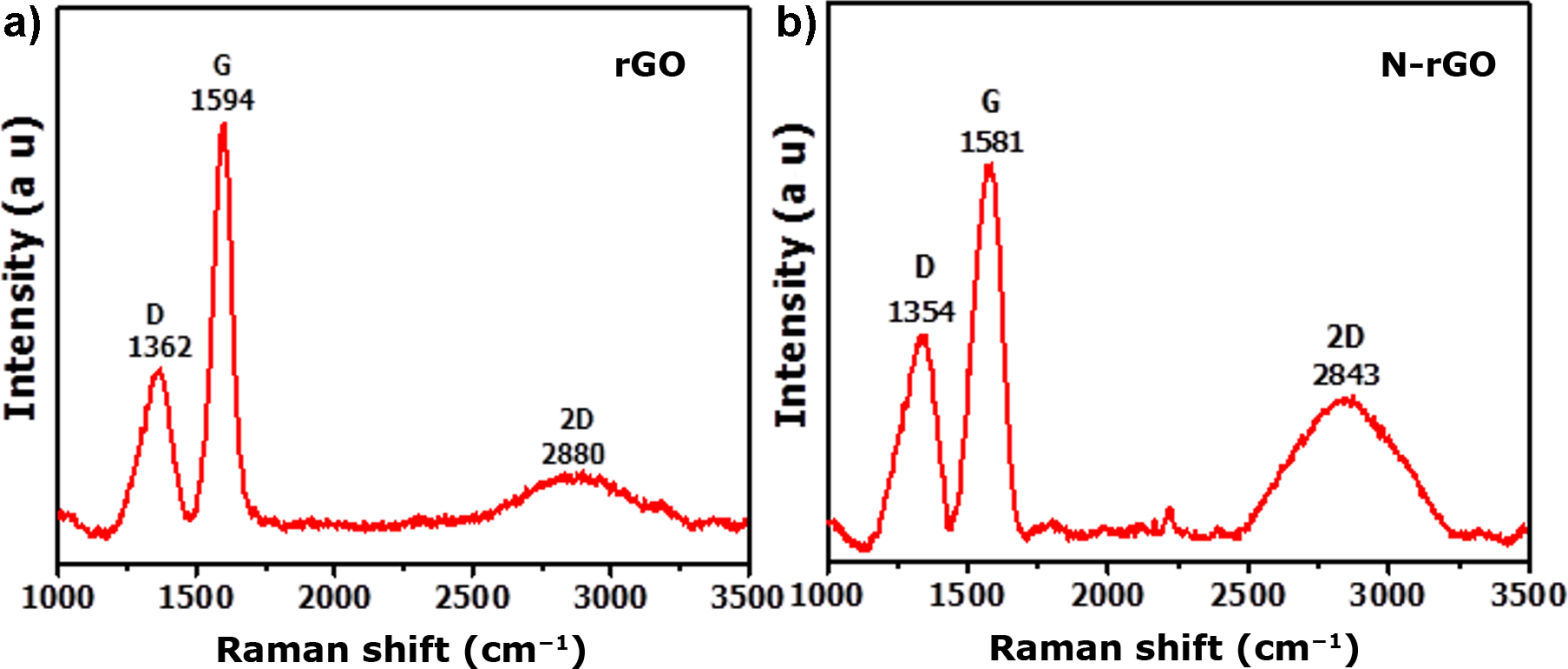
Raman spectra of (a) rGO and (b) N-rGO nanosheets.

Figure 2 shows the Raman spectra of the rGO and N-rGO nanosheets. The Raman spectrum of the rGO sample (Figure 2a) shows D, G and 2D band at, respectively, 1362, 1594 and 2880 cm^−1^. The spectrum of the N-rGO sample (Figure 2b) shows D, G and 2D band at, respectively, at 1354, 1581, and 2843 cm^−1^. The D-band is associated with the breathing modes of six-membered carbon rings that are activated by defects and structurally disordered, and the G-band originates from in-plane vibrations of sp^2^-hybridized carbon atoms in the rGO domains. The 2D-band is the second order of the D-band. The Raman results are consistent with previous reports [5].

**Figure 3:**
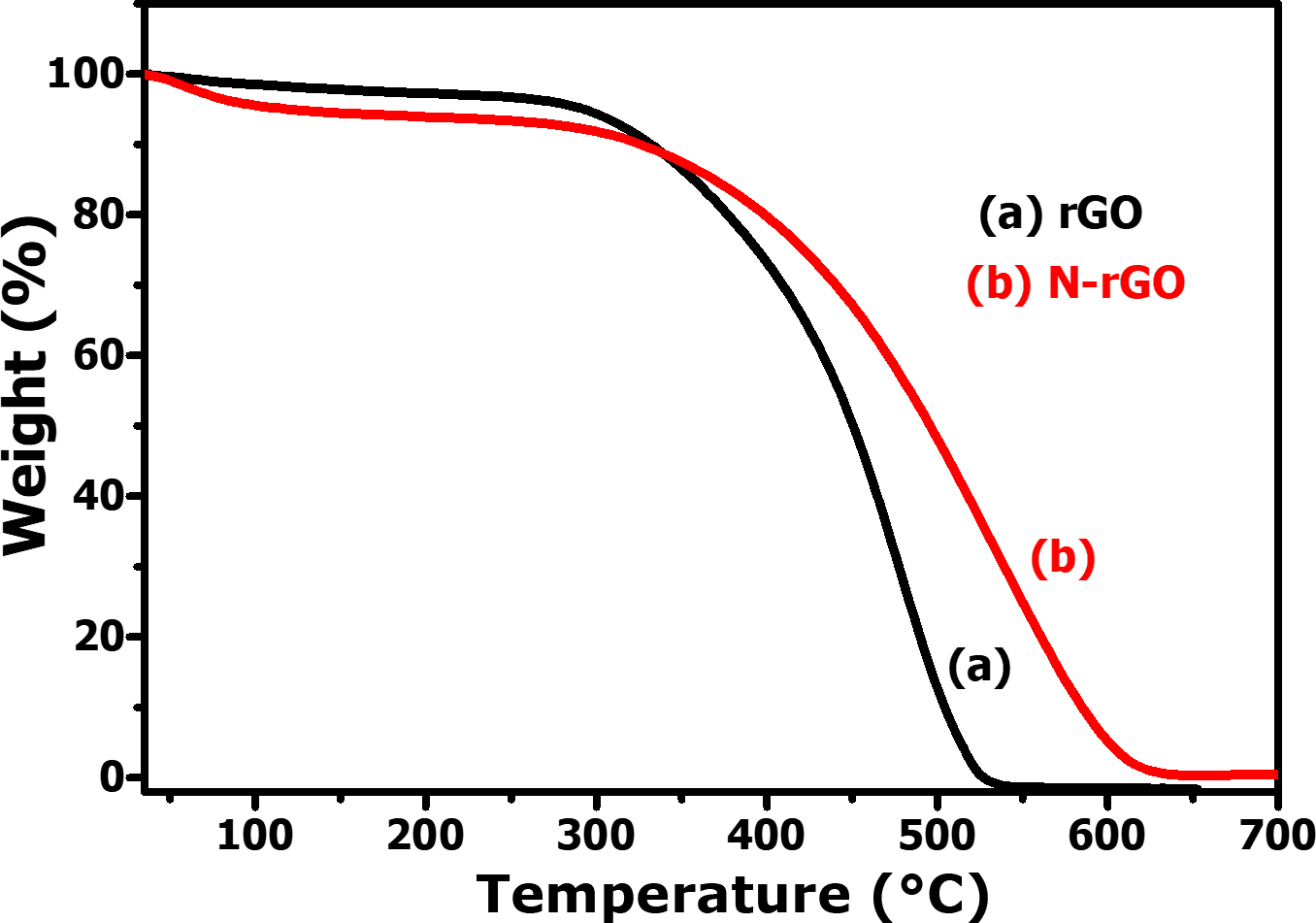
TGA curves of (a) rGO and (b) N-rGO nanosheets.

Thermogravimetric analysis (TGA) was carried out to investigate the thermal stability of the rGO and N-rGO nanosheets. The study was performed in an oxygen atmosphere at a heating rate of 3 °C·min^−1^. The results of rGO and N-rGO are shown in Figure 3a and Figure 3b, respectively. The initial weight losses occurring for rGO and N-rGO between room temperature and ca. 150 °C can be attributed to the evaporation of physically adsorbed water molecules.

The second weight loss (93.4%) for rGO occurring between 350 and 540 °C can be ascribed to the decomposition of the carbon network. For N-rGO nanosheets, a significant weight loss occurs in the temperature range between 400 and 625 °C, due to the decomposition of N-rGO. The results are consistent with the previous reports suggested in the literature [25–26].

**Figure 4:**
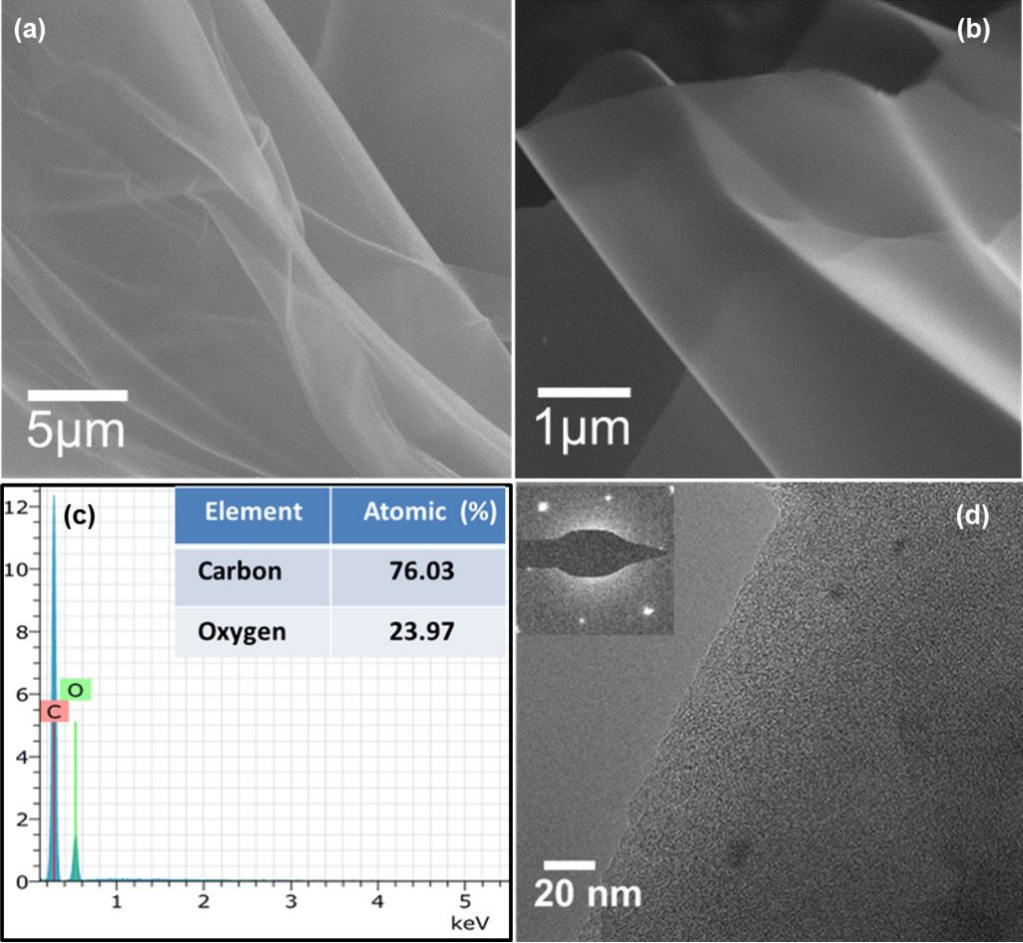
(a, b) SEM images, (c) EDS pattern and chemical composition (inset), and (d) TEM image and SAED pattern (inset) of the rGO nanosheets.

Typical SEM and TEM images of rGO at different magnifications are shown in Figure 4a,b. The low-magnification SEM image of the prepared rGO sample is composed of a large number of nanosheets, as shown in Figure 4a. The high-magnification SEM image (Figure 4b) shows that the nanosheets possess a smooth surface and are loosely stacked.

Energy-dispersive X-ray spectroscopy (EDS) was also measured to determine the chemical composition of rGO. Result from EDS shows (Figure 4c) that the product contains only C and O. The atomic fractions of C and O are found to be 76.03% and 23.97%, respectively, as shown in the inset of Figure 4c. The TEM image shown in Figure 4d indicates that the rGO sample is comprised of nanosheets with a smooth surface. The TEM image is in accordance with the SEM image (Figure 4b) of the sample. The selected area electron diffraction (SAED) pattern of the rGO sheets (inset in Figure 4d) shows a hexagonal pattern indicating the crystalline nature of the rGO sheets.

**Figure 5:**
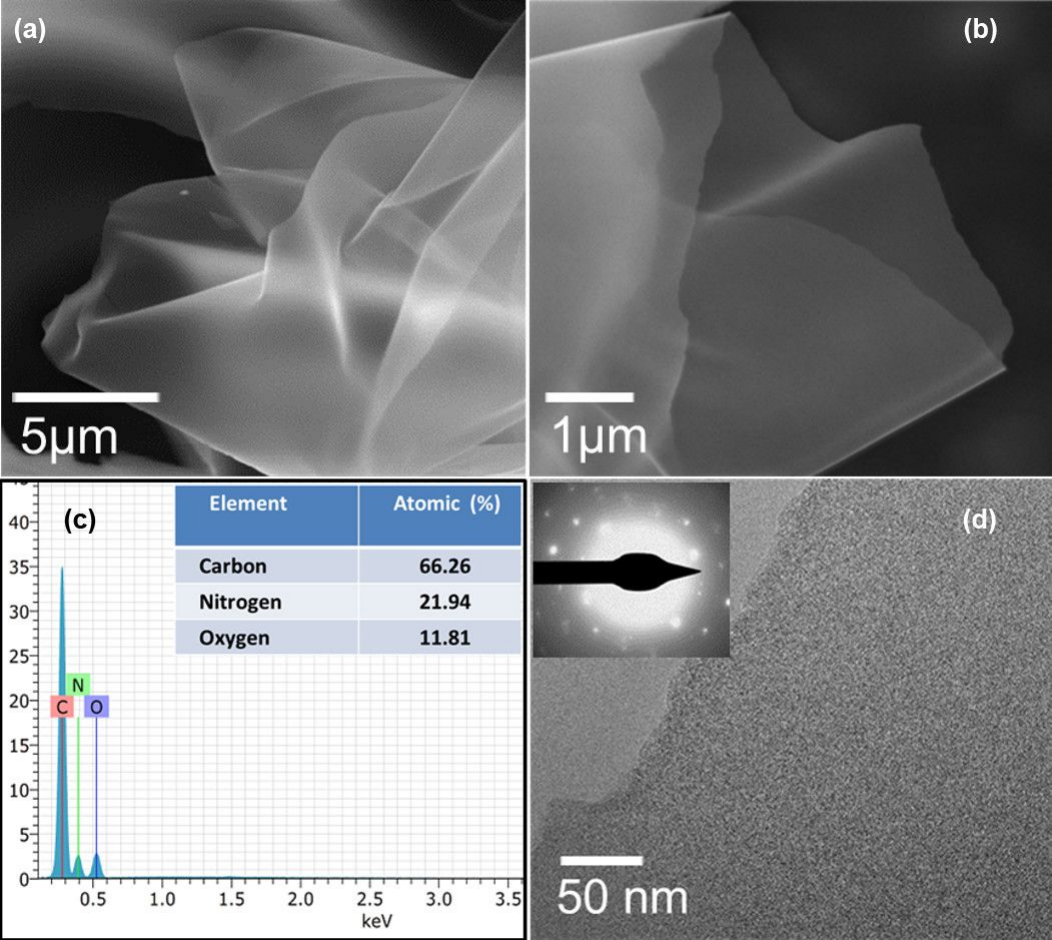
(a, b) SEM images, (c) EDS pattern and chemical composition (inset), and (d) TEM image and SAED pattern (inset) of the N-rGO nanosheets.

SEM images of N-rGO are shown in Figure 5a,b. The SEM images of N-rGO show morphological features that are similar to those of rGO. EDS shows (Figure 5c) that the product contains only C, N and O. The atomic fractions of C, N and O are found to be 66.26%, 21.94%, and 11.81%, respectively, as shown in the inset of Figure 5c. The presence of nitrogen in the as N-rGO sample is also confirmed by the estimation of N content via CHNS analysis. The result shows that the weight percentage of N element in the N-rGO nanosheets is found to be approximately 20%, and this is in good agreement with the EDS results.

The TEM image in Figure 5d clearly shows that the N-rGO sample is composed of nanosheets with a smooth surface. The SAED pattern of the N-rGO sheet (inset in Figure 5d) shows a hexagonal pattern suggesting the crystalline nature of the synthesized N-rGO sheets.

**Figure 6:**
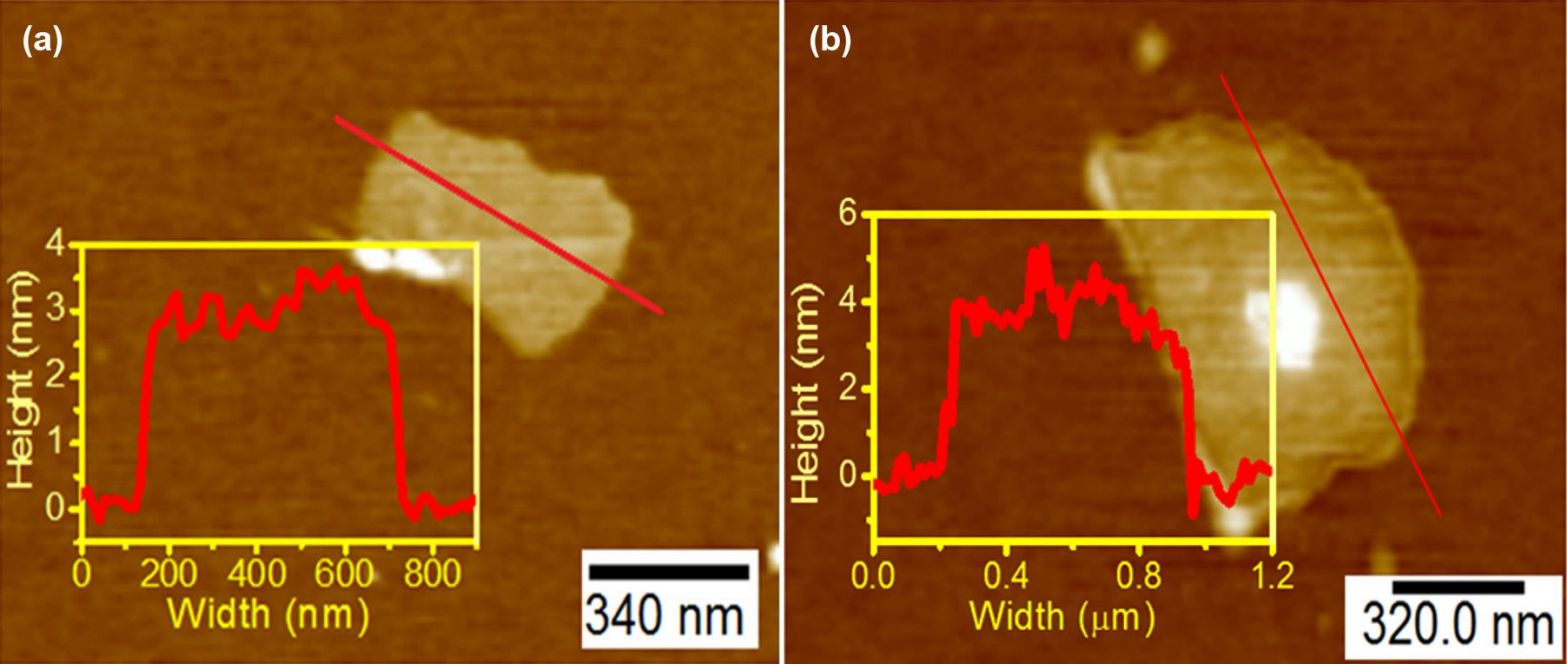
AFM images of (a) rGO and (b) N-rGO nanosheets.

AFM height images of as-prepared rGO and N-rGO nanosheets are displayed in Figure 6a and Figure 6b, respectively. The rGO and N-rGO nanosheets are flat with an average thickness of about 3 nm and 3.5 nm, respectively, with their lateral dimension in the range of several hundred nanometers.

**Figure 7:**
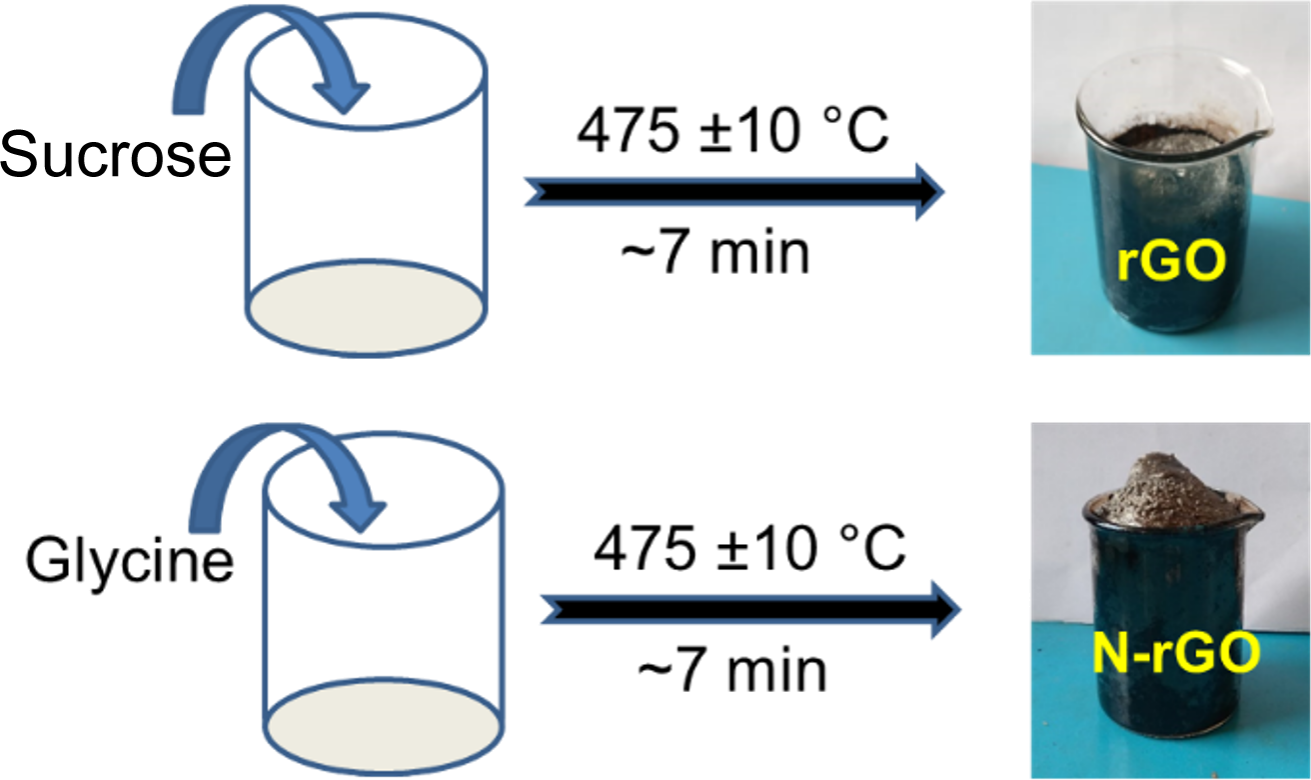
Schematic diagram of the formation of rGO and N-rGO nanosheets.

The experimental process and the digital photographs of rGO and N-rGO products are shown in Figure 7. Sucrose consists of one molecule of glucose and one molecule of fructose. The sucrose molecule contains –OH, -CH_2_OH, and –C–O–C– functional groups. At a temperature of 475 °C in a pre-heated muffle furnace, within a short period of time, sucrose undergoes covalent cross-linking reactions yielding the formation of C–C and C=C bonds through the removal of water and CO_2_. It is finally converted into a lightweight fluffy kind of material called graphene oxide nanosheets. The product was left in a furnace for ca. 7 min to get the pure phase without any impurities. The fraction of oxygen in the prepared sample is ca. 24 atom %, as measured with EDS, and the XRD pattern of the sample shows a broad peak around 23.5°. Therefore, the obtained product can be considered to be reduced graphene oxide. Similarly, glycine also undergoes a cross-linking reaction with the removal of CO_2_ and H_2_O leading to the formation of nitrogen-doped reduced graphene oxide nanosheets. For comparison we have also synthesized rGO sheets at 400 and 600 °C as well. The corresponding XRD patterns, Raman spectra and SEM images are given in Figure S1 and Figure S2, respectively, in Supporting Information File 1.

**Figure 8:**
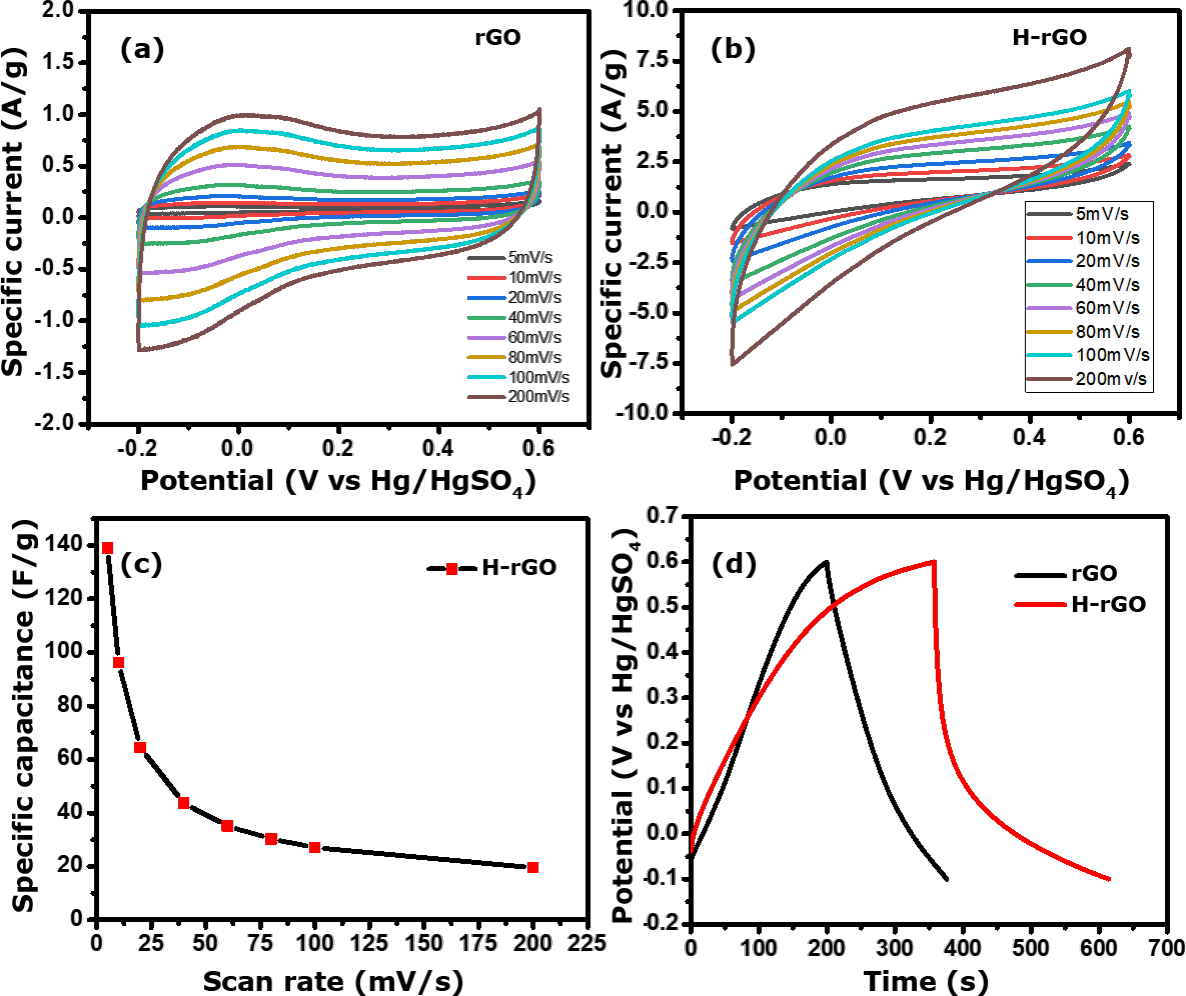
CV curves for (a) rGO and (b) H-rGO samples at different scan rates; (c) specific capacitance for H-rGO at scan rates from 5 to 200 mV·s^−1^; (d) charge–discharge curves of rGO and H-rGO at a current density of 0.5 A·g^−1^.

In order to understand the supercapacitor behavior of rGO and H-rGO (hydrogen-treated rGO) samples, we have carried out cyclic voltammetry and CD experiments with three-electrode system using 1 M H_2_SO_4_ as electrolyte (Figure 8).

**Figure 9:**
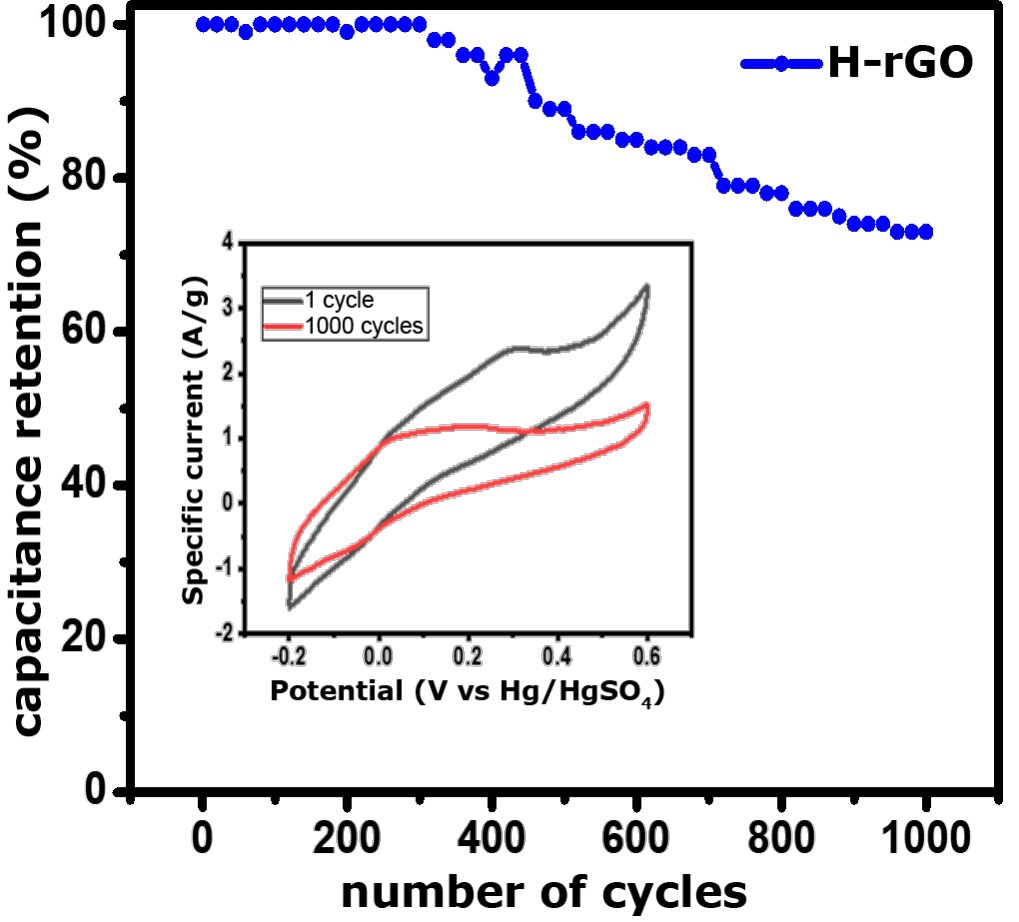
Cycling stability of H-rGO.

CV curves of rGO and H-rGO at different scan rates from 5 to 200 mV·s^−1^ vs Hg/Hg_2_SO_4_ are shown in Figure 8a and Figure 8b, respectively. These curves show increase in current density with decreasing scan rate, suggesting that the samples have ideal capacitor characteristics. However, the H-rGO sample shows a higher current density and hence a higher specific capacitance than rGO. The calculated specific capacitance values from the CV of the rGO and H-rGO electrodes at 5 mV·s^−1^ are 7 (not shown) and 139 F·g^−1^, respectively. We have checked the rate capability of the working electrode at different scan rates of 10, 20, 40, 60, 80, 100, and 200 mV·s^−1^ and observed that the specific capacitance values were 96, 64, 43, 35, 30, 27 and 19 F·g^−1^, respectively (Figure 8c). For comparison, the charge–discharge (CD) curves for rGO and H-rGO nanosheets were recorded at a current density of 0.5 A·g^−1^ in 1 M H_2_SO_4_ (Figure 8d). The specific capacitance values obtained from the CD curves at a current density of 0.5 A·g^−1^ for rGO and H-rGO were 137 and 203 F·g^−1^, respectively. The CD curve of H-rGO also shows a higher specific capacitance compared to rGO. The higher specific capacitance of H-rGO is attributed an increased conductivity due to the reduced number of functional groups after hydrogen reduction of the rGO sample. After 1000 cycles, the H-rGO sample shows 73% retention, implying that the H-rGO has excellent stability (Figure 9). We compared our result with other materials reported recently (Table 1). For conductivity measurements, the H-rGO sample was dispersed in ethanol and drop-cast on a gold gap electrode. The average resistance measured using a Keithley source meter is ca. 4 MΩ. The corresponding electrical conductivity obtained for H-rGO is ca. 0.068 S/m.

**Table 1:** Comparison of the specific capacitance of H-rGO sheets with reported values.

material	structure	specific capacitance (F·g^−1^)	reference

porous electrochemically rGO	nanosheets	81 ± 3	[27]
graphene oxide	nanosheets	121	[28]
RGO/H*_x_*PO*_y_*	nanosheets	101	[29]
RGO	nanosheets	90	[29]
H-rGO	nanosheets	139	this work

## Conclusion

In conclusion, we present a very simple and efficient method for the successful synthesis of nanosheets of rGO and of rGO with high nitrogen content by the thermal decomposition of sucrose and glycine. We measured the specific capacitance and carried out charge–discharge experiments. The rGO nanosheets that were hydrogen-treated (H-rGO) showed good supercapacitor behavior.

## Experimental

### Preparation of rGO and N-rGO nanosheets

Sucrose in the form of granulated table sugar from a retail store and analytical grade glycine from Sigma-Aldrich were purchased and used without further purification. Reduced graphene oxide (rGO) was prepared by using granulated table sugar. 2.0 g of sucrose was taken in a 100 mL borosil glass beaker. Then, the beaker was directly introduced into the preheated muffle furnace maintained at 475 ± 10 °C in oxygen atmosphere. The sugar undergoes dehydration, producing a black foam in ca. 7 min. Finally, the resultant product was collected for further analysis. A similar procedure was followed to prepare N-rGO using glycine as precursor.

### Hydrogen treatment of rGO nanosheets

Hydrogen-treated reduced graphene oxide (H-rGO) was obtained as follows. 0.5 g of the rGO sample was taken in a ceramic boat and placed in a tube furnace. The sample was heated at 700 °C for 1 h in a continuous flow of H_2_/Ar gas (5% hydrogen gas with Argon). After the reaction, the temperature was allowed to cool down to room temperature naturally. The resulting product was collected and used for the electrochemical supercapacitor measurements. The obtained results were compared with the as-synthesized rGO nanosheets.

### Materials characterization

The samples were characterized using transmission electron microscopy (TEM), atomic force microscopy (AFM), X-ray diffraction (XRD) and thermogravimetric analysis (TGA). X-ray diffraction patterns of the samples were collected in the range of 10–70° (2θ) using a Bruker D8 diffractometer with a Cu Kα source (λ = 0.154178 nm). The morphology of the samples was examined using a Tescan Mira3 field-emission scanning electron microscope (FESEM) equipped with an energy-dispersive X-ray spectroscopy (EDS). The TEM, HRTEM images and SAED patterns were obtained on a TALOS F200S G2, 200 kV FEG, and a CMOS camera (4k × 4k). The TEM samples were prepared by suspending the samples in ethanol, using an ultrasonic bath, and subsequent dripping of the suspension on the grid and drying. Raman spectra of the samples were recorded using a Jobin Yvon LabRam HR spectrometer with a 514 nm Ar laser. Thermogravimetric analysis of the samples was carried out in an oxygen flow with a heating rate of 3 °C·min^−1^ using a Mettler-Toledo-TG-850 apparatus. AFM measurements were performed using a CP2 atomic force microscope.

### Electrode preparation and electrochemical characterization

The catalyst inks of as-synthesized rGO and reduced graphene oxide H-rGO were prepared by ultrasonication separately. A mixture of 4.0 mg rGO and 0.025 wt % (5 μL) of Nafion in 0.4 mL of dimethylformamide (DMF) was sonicated until a homogeneous dispersion was obtained. 3 μL catalyst ink was taken and drop-cast onto a glassy carbon electrode, which was allowed to dry at room temperature. A similar procedure was followed to prepare the H-rGO electrode. The electrochemical studies, including cyclic voltammetry (CV) and chronopotentiometry charge–discharge (CD), were carried out at room temperature in 1 M H_2_SO_4_ solution in a standard three-electrode cell using an electrochemical workstation CHI 660E. This system consists of a glassy carbon working electrode (3 mM), a platinum wire counter electrode and a Hg/Hg_2_SO_4_ reference electrode with 1 M H_2_SO_4_ electrolyte. The specific capacitance (SC) of rGO and H-rGO was calculated from CV curves, according to Equation 1:

[1] SC=∫IdV/2νm⋅ΔV,

where ∫*I*d*V* is the area under the CV curve, *m* is the mass of the active material, ν is the potential scan rate (V/s), and Δ*V* is the potential window. We have also calculated the specific capacitance from CD curves using Equation 2:

[2] SC=It/m⋅ΔV,

where *I* is the current, *t* is the time of the discharge cycle, *m* is the mass of the active material, and Δ*V* is the potential window of the discharge cycle.

## Supporting Information

File 1 Additional experimental data.
