# Supplementary material for: Simple synthesis of nanosheets of rGO and nitrogenated rGO
Source: Beilstein J Nanotechnol. 2020 Jan 7;11:68–75. doi: 10.3762/bjnano.11.7 (PMC6964660; doi:10.3762/bjnano.11.7)
Supplement: File 1 — Additional experimental data. [file Beilstein_J_Nanotechnol-11-68-s001.pdf]

BEILSTEIN JOURNAL OF NANOTECHNOLOGY

Supporting Information

for

Simple synthesis of nanosheets of rGO and nitrogenated rGO

Pallellappa Chithaiah, Madhan Mohan Raju, Giridhar U. Kulkarni and C. N. R. Rao

Beilstein J. Nanotechnol. **2020,** *11,* 68–75. doi:10.3762/bjnano.11.7

Additional experimental data

License and Terms: This is a supporting information file under the terms of the Creative Commons Attribution License (https://creativecommons.org/licenses/by/4.0). Please note that the reuse, redistribution and reproduction in particular requires that the authors and source are credited.
The license is subject to the *Beilstein Journal of Nanotechnology* terms and conditions: (https://www.beilstein-journals.org/bjnano)

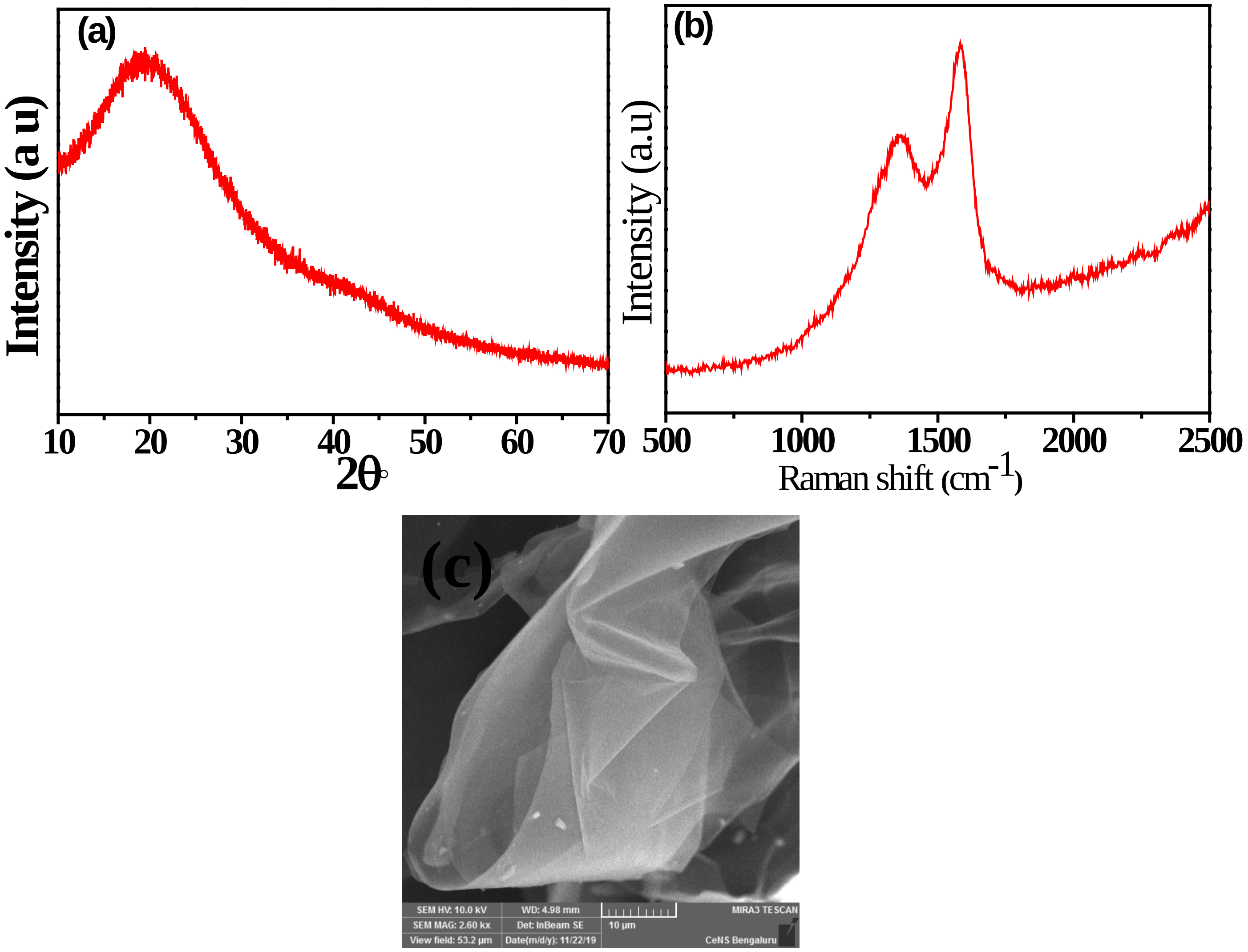

Figure S1: (a) XRD pattern, (b) Raman spectrum, and (c) SEM image of rGO nanosheets synthesized at 400 °C.

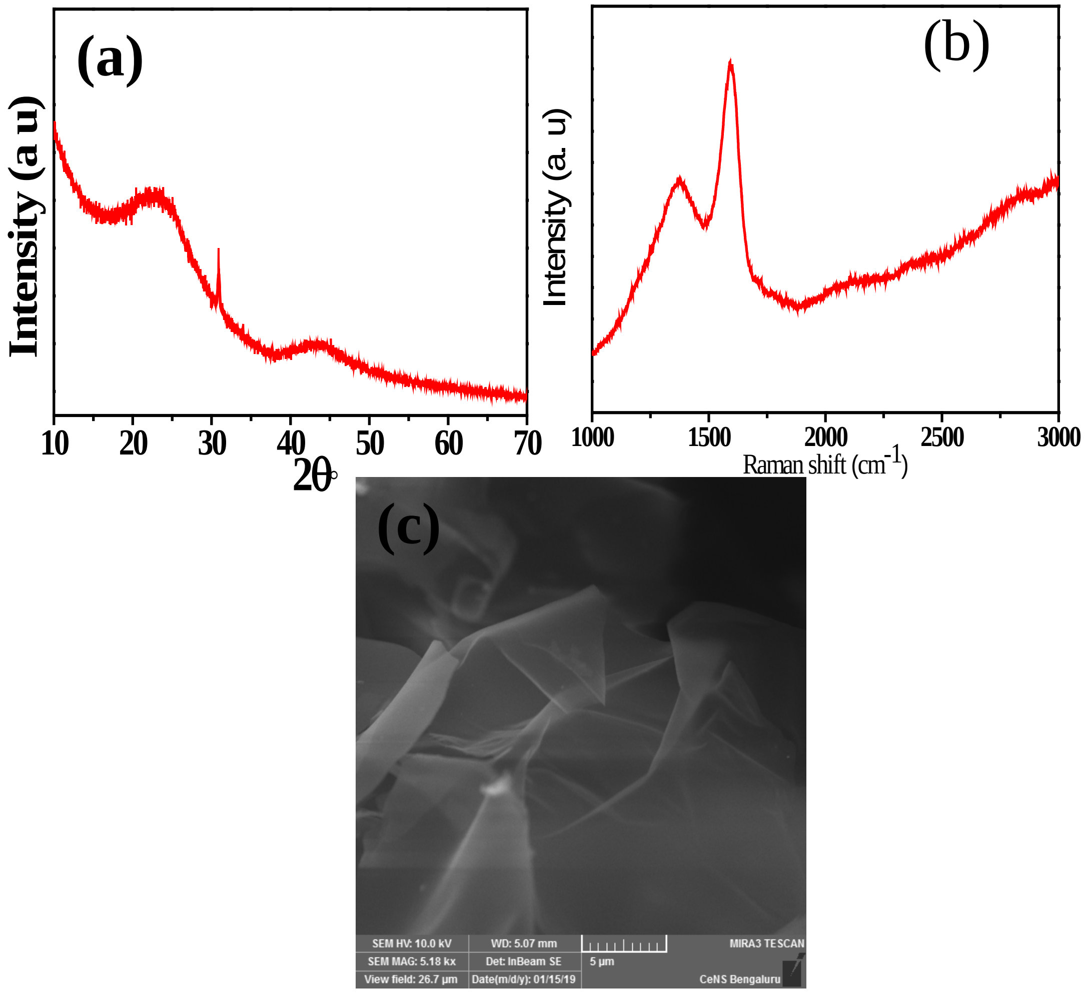

Figure S2: (a) XRD pattern, (b) Raman spectrum, and (c) SEM image of rGO nanosheets synthesized at 600 °C.

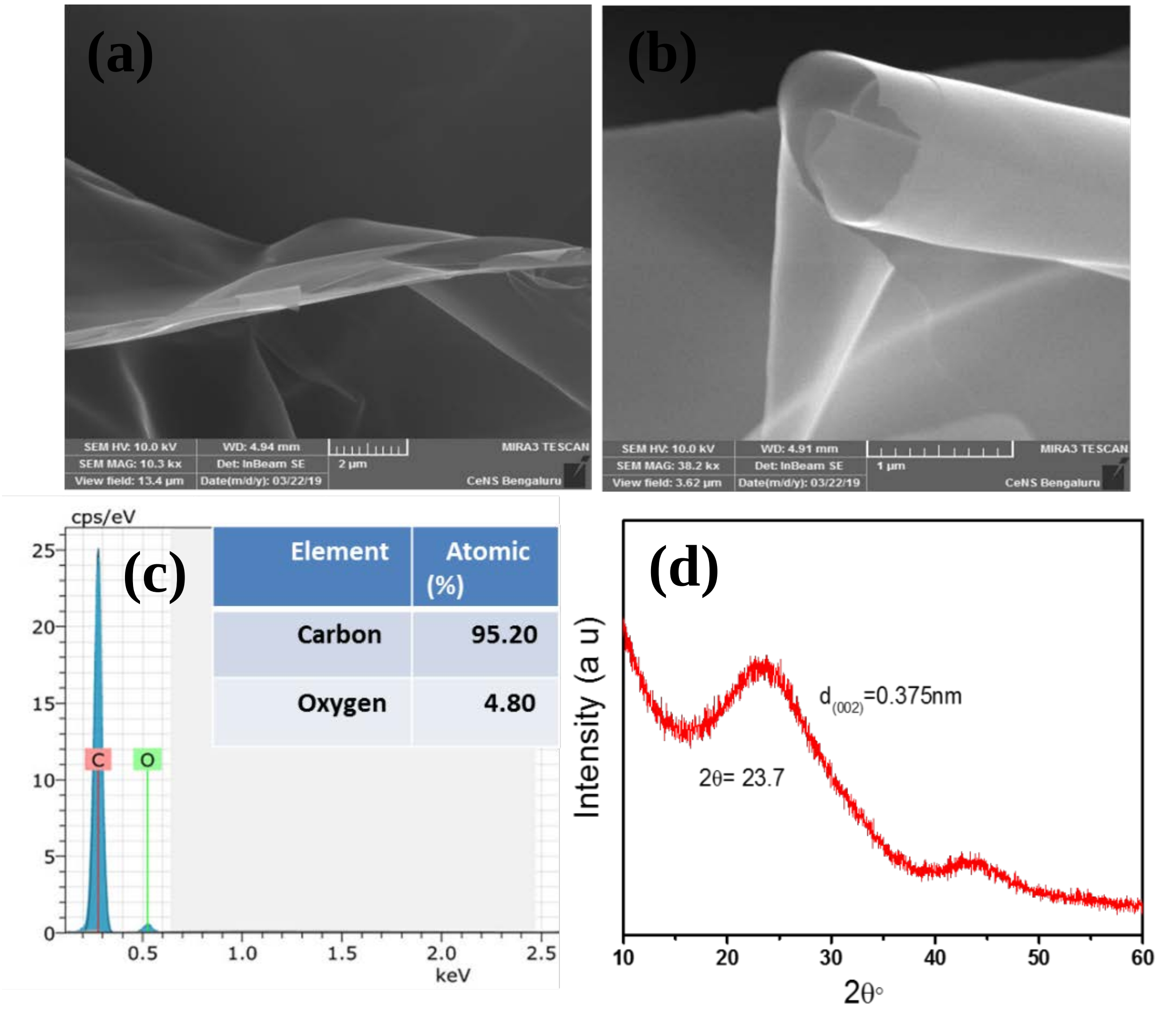

Figure S3: (a, b) SEM images, (b)EDS spectrum (inset: chemical composition), and (c) XRD pattern of H-rGO nanosheets.
